# Gender Differences in Knowledge, Attitudes, and Practices toward COVID-19 in Saudi Arabia: A Cross-Sectional Study

**DOI:** 10.3390/diseases11020063

**Published:** 2023-04-19

**Authors:** Mohammed Saif Anaam, Saud Alsahali

**Affiliations:** 1 Department of Pharmacy Practice, Unaizah College of Pharmacy, Qassim University, P.O. Box 5888, Unaizah 51911, Saudi Arabia; 2 Al-Rowaad Medical College, Department of Pharmacy Practice, P.O. Box 8892, Sana’a 5696, Yemen

**Keywords:** COVID-19, attitudes, knowledge, practices, Saudi Arabia

## Abstract

Days after the World Health Organization (WHO) declared that COVID-19 was a pandemic, Saudi Arabia took preventative and precautionary measures to avoid its spread and to safeguard its citizens. In this study, we investigated the knowledge, attitudes, and practices (KAP) of both men and women toward COVID-19 as well as associated factors. A cross-sectional study was conducted using an online, self-report questionnaire distributed via Google Forms. The overall percentage of correct answers for the knowledge statements was 80.2%, with a higher rate among the female respondents (82.4% vs. 78.5%, *p* = 0.005). Slightly more than half (i.e., 165: 51.6%) of the participants showed that they did not go to crowded places during the pandemic; however, more female respondents recorded that they avoided crowded places than male respondents (57.7% vs. 46.2%, *p* = 0.04). Most participants (i.e., 272: 85.0%) reported that they had worn a mask in recent days, and more than two-thirds (84.4%) said that they still follow the strategies recommended by government authorities to prevent the spread of the virus. Again, more female respondents reported this than males (89.9% vs. 79.5%; *p* = 0.01). Significant correlations (*p* < 001) were noted between knowledge and practices (r = 0.31), knowledge and attitudes (r = 0.37), and attitudes and practices (r = 0.29). In the multivariate logistic regression analysis, occupation and education were independently associated with knowledge among both the male and female respondents (adjusted odds ratio [aOR]: 2.9; 95% confidence interval [CI]: 1.2–7.2; aOR: 5.9; 95% CI: 2.2–15.9). Residence was independently associated with attitudes, but only among the male respondents (aOR: 2.3; 95% CI: 1.1–4.9), and COVID-19 was independently associated with practices among both the male and female respondents (aOR: 4.5; 95% CI: 1.4–14.2; aOR: 9.8; 95% CI: 1.2–81.2). There were significant gender differences in both knowledge and practices toward COVID-19, with the female respondents achieving better scores than the male respondents. Thus, we recommend that health education campaigns are tailored to specifically target males.

## 1. Introduction

The novel coronavirus (2019-nCoV), now called severe acute respiratory syndrome coronavirus (SARS-CoV-2 or COVID-19), was first reported in the city of Wuhan, China, in December 2019, after the detection of many pneumonia cases of unknown etiology [1,2,3]. It spread rapidly to almost all countries in the world, threatening the lives of millions of people. On 30 January 2020, the World Health Organization (WHO) declared the outbreak a public health emergency of global concern [4]. After a fast and steep increase in reported cases worldwide, the WHO announced that the COVID-19 outbreak was a pandemic on 11 March 2020 [5]. Although the coronavirus can infect people of all age groups, older people and those with pre-existing medical conditions are more vulnerable [6]. The symptoms of COVID-19 are similar to those of flu, including fever, dry cough, fatigue, myalgia, shortness of breath, and dyspnea [7,8]. In some severe cases, it can cause respiratory failure, cardiac complications, and death [9]. In Saudi Arabia, the first case of COVID-19 was reported on 2 March 2020, by a citizen who had returned from Iran via the Kingdom of Bahrain [10]. Subsequently, the Saudi government implemented many proactive and precautionary measures to decrease the rate of COVID-19’s spread. These proactive steps included restrictions on both domestic travel and international travel; shopping center closures; enforced lockdowns; the implementation of curfews during most of the day; the suspension of educational activities in academic and religious institutions (e.g., universities and congregational prayers at mosques, including the two holy mosques in Mecca and al-Madinah); and a decrease in the number of employees in workplaces, except for essential and basic services [11]. The educational activities in schools and universities across the country were shifted to online learning. Additionally, the companies and governmental services facilitated working from home for their staff by using technology [11]. These unprecedented preventative measures, which were applied at the national level to control the pandemic, although successful, affected both the social and economic life of the people mainly through restricting mobility, as expected and documented in the literature [12]. However, it is still necessary to consider public behavior and knowledge regarding COVID-19 because of intensified public (non-)adherence to the measures established by health authorities. The public’s adherence to the measures is expected to be affected by knowledge and attitudes toward COVID-19 among the general population. The results of many studies showed the importance of public knowledge in controlling a pandemic [13,14]. Investigating public knowledge concerning COVID-19 may lead to a better understanding of existing public perceptions and practices. This would help to identify the attributes that influence the public to adopt healthy practices and responsive behaviors [15]. Assessing public knowledge in the general population is important in pinpointing gaps and in strengthening ongoing preventive efforts, as many published studies have investigated [16,17,18,19]. Because gender can influence people’s behavior, it can, consequently, affect individuals’ health [16]. Given this, we felt that it was important to focus on and assess this factor; therefore, in this study, we aimed to investigate the gender-related differences in people’s knowledge, attitudes, and practices (KAP) regarding COVID-19 in Saudi Arabia. 

## 2. Methodology

### 2.1. Study Design and Sampling

This cross-sectional study was conducted among the general population of Saudi Arabia between 13 October and 13 December 2021. This method was used because it is considered a suitable method for investigating KAP either at a local or national level. In this study, we used an online, self-report questionnaire from Google Forms for data collection. Using Raosoft software [20] for sample size calculation, 95% was considered as the confidence level, and 5% as the margin of error. A response rate of 76.1% was used, as reported in a previous study [21]. According to the latest Saudi census, the population size is 35,013,414 [22]. The minimum sample size was estimated to be 280. Accounting for 30% of expected not responses, the required sample size was 364. By the end of the designated study period (i.e., 13 December 2021), 320 questionnaires had been obtained, with a response rate of 87.9%. 

### 2.2. Administration of the Questionnaire

A questionnaire from a previously published study [11] was adapted, with permission from the main author. The suitability of the draft instrument was examined for estimating validity (content and face) and reliability. For content validity, the first draft was presented to a panel of three academic staff who independently examined the instrument’s content and question format, as well as the sequencing and clarity of the questionnaire as a whole, and no modifications were made. Regarding the face validity, the draft was administered to 10 people, and time was taken to complete the questionnaire; difficulties in participants’ comprehension and the extent of participant acceptance were recorded. No comments were made by the participants. The Cronbach’s alpha for the KAP questionnaire was 0.776, which is considered acceptable and consistent [23]. The questionnaire was distributed to the public via WhatsApp and Twitter. The research team sent several reminders to encourage all respondents to complete the survey. To avoid duplicate responses from the same respondent, we consider only one response from the same device. In the first part of the questionnaire, there were eight questions about demographic data. Then, the second part contained 12 statements that assessed the participants’ knowledge of COVID-19. The true statements were given a score of 1, and false statements were given a score of 0. The highest attainable knowledge score was 12. There were three questions on attitudes in the third part and three questions on practices in the fourth part. The six questions on attitudes and practices were framed using three possible answers (*yes*, *no*, and *not sure*). A score of 1 was given for each positive reaction toward the questions on attitudes and practices, and a score of 0 was assigned to a negative reaction. *Not sure* was recorded as a negative reaction. The maximum score for attitudes and practices was 3. Mean scores were used as cutoff points to assess KAP levels. A brief explanation of the study’s objectives and benefits, emphasizing the confidentiality and use of personal data for scientific work, was provided to the participants through an invitation message, sent via WhatsApp. Consent to participate was obtained once the participants responded to the online survey. Participation in this study was completely voluntary.

### 2.3. Data Analysis

The Statistical Package for Social Sciences software (SPSS, version 26; SPSS, Chicago, IL, USA) was used for data analysis. Descriptive statistics, including frequencies, percentages, means, and standard deviations, were used to summarize the participants’ responses to the KAP statements. Pearson’s chi-square test was employed to examine the associations between the categorical variables. An independent *t*-test was used to study gender differences in the means of continuous variables. Logistic regression analysis was performed to predict factors associated with KAP. The Kolmogorov–Smirnov (K-S) test was performed to test the normal distribution of continuous variables before we conducted inferential statistical tests. A *p*-value of <0.05 was considered statistically significant.

### 2.4. Ethical Approval

This study was conducted in accordance with the Declaration of Helsinki and approved by the Committee of Health Research Ethics of the Deanship of Scientific Research, at Qassim University (approval #21-04-21).

## 3. Results

### 3.1. Demographic Characteristics

A total of 320 individuals participated in the study. Their mean (±SD) age was 27.4 ± 10.2, and the majority were male (53.4%). Most participants (i.e., 225: 79.7%) were in the younger age group (18–30 years old). Approximately two thirds (74.4%) were unmarried and 8.1% reported that they had contracted COVID-19 at some point. The vast majority of participants, specifically, 301 (94.1%), did not have any coexisting diseases. Their demographic characteristics are shown in Table 1.

### 3.2. Knowledge of COVID-19

The overall percentage of correct answers for the knowledge statements was 80.2%, with a higher rate among females (82.4% vs. 78.5%, *p* = 0.005). Table 2 shows the detailed responses to the knowledge statements. A statistically significant association between gender and knowledge was observed in the responses to questions six (*p* = 0.01) and seven (*p* = 0.04). The *t*-test showed a statistically significant difference (*p* < 0.05) in the means of the knowledge scores: the mean scores of the female and male respondents were 9.89 (±2.14) and 9.42 (±2.30), respectively. In the multivariate logistic regression analysis, occupation was independently associated with knowledge among the male respondents (aOR: 2.9; 95% CI: 1.2–7.2), while education was independently associated with knowledge among the female respondents (aOR: 5.9; 95% CI: 2.2–15.9).

### 3.3. Attitudes toward COVID-19

Slightly more than two thirds (78.7%) of the participants answered the questions regarding attitudes positively. The total score for attitudes was three, while the mean score was 2.36 ± 0.81. More than two thirds of the participants (78.8%) agreed that COVID-19 would eventually be successfully controlled. Approximately 68.0% believed that COVID-19 was a health threat to the community. The vast majority (89.4%) believed that lockdowns would improve society’s overall well-being, in terms of controlling the COVID-19 pandemic. Table 3 summarizes the responses to the questions on attitude by gender. The *t*-test failed to show a statistically significant difference in the mean attitude scores between the male (2.32 ± 0.88) and female (2.42 ± 72) respondents. However, there was a statistically significant difference in the mean knowledge scores between the male and female respondents in the three questions on attitudes (Table 4). In the multivariate logistic regression, residence was independently associated with positive attitudes, but only among the male respondents (aOR: 2.3; 95% CI: 1.1–4.9).

### 3.4. Practices toward COVID-19

Approximately two-thirds (73.7%) of the sample reported good practices regarding the COVID-19 pandemic. The total score for practices was three, while the mean score was 2.21 ± 0.84. The study revealed non-satisfactory adherence to preventive measures, such as avoiding crowded places, in both the male and female respondents, with 53.8% and 42.3% non-adherence reported, respectively. The responses to the questions about practice are presented in Table 5. A chi-square analysis of the two practice indicators (i.e., not going to crowded places during the pandemic and following the strategies recommended by the authorities to prevent infections and the spread of COVID-19) demonstrated a statistically significant association between gender and practices (*p* < 0.05). The *t*-test indicated a statistically significant difference (*p* = 0.004) in the mean scores for practices between both the male (2.08 ± 0.90) and female respondents (2.36 ± 0.75). There was also a statistically significant difference in the mean knowledge scores between both the male and female respondents for all three questions about practices (Table 6). Moreover, the multivariate logistic regression analysis illustrated that having contracted COVID-19 was independently associated with good practices among both the male (aOR: 4.5; 95% CI: 1.4–14.2) and female respondents (aOR: 9.8; 95% CI: 1.2–81.2).

### 3.5. Correlations between the Scores

The Pearson’s correlation test showed that there is a statistically significant association between knowledge, attitudes, and practices (*p* < 0.001). The highest correlation coefficient was found between knowledge and attitudes (0.37), followed by (0.31) the relationship between practices and knowledge, and (0.29) the relationship between attitudes and practices.

## 4. Discussion

Gender influences behavior and, therefore, affects people’s health. It also affects the roles and responsibilities of men and women. Women care more about their health and, consequently, give more consideration to health issues than men [16]. The present study examined gender differences in the knowledge, attitudes, and practices regarding COVID-19 among the 320 participants who took part in the survey. A good level of knowledge about COVID-19, with an overall rate of 80.2%, and with a higher rate among the female respondents, was observed (82.4% vs. 78.5%, *p* = 0.005). Most of the participants gave correct answers in response to most of the knowledge statements. The accuracy rate ranged from 73.7% to 92.4% among the male respondents and from 77.9% to 96% among the female respondents. The findings were in agreement with those of previous studies that have been conducted in countries such as Sudan (78.2%, 84.7%) [24,25], Jordan (80.0%) [26], Malaysia (80.5%) [27], India (81.0%) [28], Egypt (70.2%) [29], Bangladesh (69.8%, 85.0%) [30,31], Nigeria (65.4%) [32], Liberia (51.0%) [33], Cameroon (84.2%) [34], Saudi Arabia (89.9%) [35], China (91.2%) [36], and Uganda (93.9%) [37]. However, a poor response to the knowledge-related question on symptoms was a concern in the current study: only 51.0% of the male respondents and 54.4% of the female respondents answered this question correctly. These findings were in line with other studies conducted in Bangladesh [38], Ethiopia [39], and Saudi Arabia [40], in which only 55.3%, 53.3%, and 58.1% of the participants, respectively, responded to the questions correctly. We are not certain how public health preventative measures were implemented in the countries that recorded low scores regarding the knowledge of COVID-19; however, the high knowledge scores reported in the current study might be attributed to the fact that public health messages in Saudi Arabia are disseminated at the lowest administrative level, which makes them more effective. A significant difference was found in the knowledge scores between the male and female respondents in this study. This was associated with occupation and education among both the male and female respondents. These findings were similar to those in other studies [17,41,42,43,44,45,46]; however, they contrasted with the outcomes of a study performed in Pakistan [47]. The findings in our study showed a high mean score for attitudes (78.7%). This finding was comparable to the results reported in other studies [46,48,49,50,51]. Approximately 68.0% of the respondents in our study believed COVID-19 to be a serious disease that poses a health threat to their community; therefore, 89.4% believed that lockdowns and curfews during the first days of the pandemic were required to control it. However, they also thought these measures could harm the economy, mainly by affecting mobility, which is the bloodstream of business [12]. This was similar to the findings of prior studies, which have been conducted in Saudi Arabia [17,19,52,53], India [28,54,55], Bangladesh [38], Pakistan [56], China [36], Nepal [57], and those conducted in eight Western countries [58]. According to our results, Saudi people showed a generally optimistic attitude toward the COVID-19 pandemic: more than two-thirds (78.8%) of the respondents believed that the COVID-19 crisis would finally be successfully controlled. Although most participants believed COVID-19 to be a serious disease, they also knew that avoiding crowded places and gatherings would help to control the rate of infection. Despite this, however, more than half of the male respondents (53.8%) said that they went to crowded places. Such risky behaviors were seen to be related to gender, education, and occupation in the present study. As suggested by findings of published studies on the age and gender patterns of risky behaviors [59,60,61], men and late adolescents are more likely to engage in such conduct. In line with these findings, a significant association between being male and being involved in potentially dangerous practices regarding COVID-19 was shown in the present study. Muslim communities tend to encourage women to stay at home, which may have contributed to the significantly higher level of risk-taking behaviors among the male respondents in this study, e.g., going to crowded places and spending time outside of the home with friends. In addition, the widespread belief among people in Arab/Muslim countries that COVID-19 is a political issue rather than a real health issue may also be associated with this finding. A significantly positive attitude toward COVID-19 was reported among the married respondents in this study, which was in agreement with a study conducted in Jordan [36,44]. This might be because, in Arab/Muslim communities, married individuals tend to behave in a more responsible manner toward both themselves and their community, compared to single people. However, there was no significant difference between the genders in their attitudes toward COVID-19. This was in line with some past research studies [35,36,47] but in contrast to other studies [19,33,37,62]. Regarding practices, the findings revealed that good practices were associated with female respondents. Again, this was in agreement with most of the prior research studies [18,19,35,37,46,62,63], but in contrast to one study performed in Uganda [64]. It seems that the generally positive attitudes (78.7%) and good practices (73.7%) regarding COVID-19 among the individuals who participated in this study can most likely be explained by the Saudi government’s high commitment to adopting strict preventative and control measures to stop the spread of the virus. The current study suggested that there was a statistically significant correlation among the knowledge, attitudes, and practices toward COVID-19. These findings were in agreement with those reported in much of the literature [35,36,38,48,49].

### Strengths and Limitations

This study was one of the few studies that specifically aimed to address gender-related differences in people’s knowledge, attitudes, and practices toward COVID-19 in the country. However, it had some limitations. First, it lacked generalizability to the overall Saudi population, as most participants were from the Qassim region. Second, the self-report survey may have caused social desirability bias. This may have caused the participants to answer the questions about attitudes and practices positively, based on what they thought was expected of them.

## 5. Conclusions

Our findings indicated that both men and women have a good level of knowledge and display good practices toward COVID-19; however, this was especially true among the female respondents. Although there was no significant difference in the attitudes between the male and female respondents in our study, the female respondents still recorded better attitudes. In addition to theoretical implications, these findings could encourage policymakers to conduct health education campaigns and awareness events that specifically target males to further improve KAP during a crisis. However, it is considered that the goal of this study was considered achieved, as the KAP was revealed, together with recommending suitable mitigating actions. Nevertheless, further research is needed to comprehensively address the economic impact of these compact measures and to propose plans to handle impacted sectors to deal with future pandemics based on the obtained findings.

## Figures and Tables

**Table 1:** Socio-demographic characteristics (n = 320).

Variable	Male	Female	Total (%)
Gender	171 (53.4)	149 (46.6)	320 (100.0)
Mean Age (±SD)	27.9 (±10.6)	26.8 (±9.9)	27.4 (±10.2)
Age (year)			
Young Adults (18–30 year)	134 (78.4)	121 (81.2)	225 (79.7)
Middle-aged Adults (31–45 year)	20 (11.7)	15 (10.1)	35 (10.9)
Old-aged Adults (above 45 year)	17 (9.9)	13 (8.7)	30 (9.4)
Education			
School	43 (25.1)	28 (18.8)	71 (22.2)
University	128 (74.9)	121 (81.2)	249 (77.8)
Place of residence			
Qassim	129 (75.4)	99 (66.4)	228 (71.3)
Other	42 (24.6)	50 (33.6)	92 (28.8)
Occupation			
Employed	50 (29.2)	21 (14.2)	71 (22.3)
Unemployed	121 (70.8)	127 (85.8)	248 (77.7)
Marital status			
Unmarried	126 (73.7)	112 (75.2)	238 (74.4)
Married	45 (26.3)	37 (24.8)	82 (25.6)
Had COVID-19			
Yes	15 (8.8)	11 (7.4)	26 (8.1)
No	156 (91.2)	138 (92.6)	236 (91.9)
Coexisting disease			
Yes	10 (5.8)	9 (6.0)	19 (5.9)
No	161 (94.2)	140 (94.0)	301 (94.1)

**Table 2:** Gender responses to COVID-19 knowledge statements (n = 320).

Item.	Male (%)	Female (%)	Total (%)	*p* Value *
K1. The main clinical symptoms of COVID-19 are fever, fatigue, dry cough, and muscle pain.				NS
Yes	149 (87.1)	138 (92.6)	287 (89.7)	
No/Don’t Know	22 (12.9)	11 (7.4)	33 (10.3)	
K2. Unlike the common cold, stuffy nose, runny nose, and sneezing are less common in persons infected with the COVID-19 virus.				NS
Yes	93 (54.4)	76 (51.0)	169 (52.8)	
No/Don’t Know	78 (45.6)	73 (49.0)	151 (47.2)	
K3. Currently there is no effective cure for COVID-19, but early symptomatic and supportive treatment can help most patients to recover from the infection.				NS
Yes	126 (73.7)	121 (81.2)	247 (77.2)	
No/Don’t Know	45 (26.3)	28 (18.8)	73 (22.8)	
K4. Not all persons with COVID-19 will develop severe cases. Those who are elderly, have chronic illnesses, and are obese are more likely to be severe cases.				NS
Yes	141 (82.5)	132 (88.6)	273 (85.3)	
No/Don’t Know	30 (17.5)	17 (11.4)	47 (14.7)	
K5. Eating or contacting wild animals would result in the infection by the COVID-19 virus.				NS
No	111 (64.9)	97 (65.1)	208 (65.0)	
Yes/Don’t Know	60 (35.1)	52 (34.9)	112 (35.0)	
K6. Persons with COVID-19 cannot spread the virus to others when the symptoms of COVID-19 are not present.				0.01
No	111 (64.9)	116 (77.9)	227 (70.9)	
Yes/Don’t Know	60 (35.1)	33 (22.1)	93 (29.1)	
K7. The COVID-19 virus spreads via respiratory droplets of infected individuals.				0.04
Yes	149 (87.1)	140 (94.0)	289 (90.3)	
No/Don’t Know	22 (12.9)	9 (6.0)	31 (9.7)	
K8. Ordinary individuals can wear general medical masks to prevent the infection by the COVID-19 virus.				NS
Yes	157 (91.8)	133 (89.3)	290 (90.6)	
No/Don’t Know	14 (8.2)	16 (10.7)	30 (9.4)	
K9. It is not necessary for children and young adults to take measures to prevent the infection by the COVID-19 virus.				NS
No	153 (89.5)	134 (89.9)	287 (89.7)	
Yes/Don’t Know	18 (10.5)	15 (10.1)	33 (10.3)	
K10. To prevent the infection by COVID-19, individuals should avoid going to crowded places and avoid gatherings.				NS
Yes	158 (92.4)	143 (96.0)	301 (94.1)	
No/Don’t Know	13 (7.6)	6 (4.0)	19 (5.9)	
K11. Test, Trace and Isolate (TTI) are the effective ways to reduce the spread of COVID-19.				NS
Yes	143 (83.6)	129 (86.6)	272 (85.0)	
No/Don’t Know	28 (16.4)	20 (13.4)	48 (15.0)	
K12. People who have contact with someone infected with the COVID-19 virus should be immediately isolated in a proper place. In general, the observation period is 14 days.				NS
Yes	157 (91.8)	139 (93.3)	296 (92.5)	
No/Don’t Know	14 (8.2)	10 (6.7)	24 (7.5)	

* chi-square test; NS—not significant.

**Table 3:** Gender attitudes towards COVID-19 (n = 320).

Item	Male (%)	Female (%)	Total (%)	*p* Value *
A1. Do you agree that COVID-19 will finally be successfully controlled?				NS
Yes	134 (78.4)	118 (79.2)	252 (78.8)	
No/Unsure	37 (21.6)	31 (20.8)	68 (21.3)	
A2. Do you think that COVID-19 is a threat for your community?				NS
Yes	114 (66.7)	104 (69.8)	218 (68.1)	
No/Unsure	57 (33.3)	45 (30.2)	102 (31.9)	
A3. I think that the lockdown would improve the overall wellbeing of the society in terms of controlling COVID-19 pandemic situation.				NS
Yes	148 (86.5)	138 (92.6)	286 (89.4)	
No/Unsure	23 (13.5)	11 (7.4)	34 (10.6)	

* chi-square test; NS—not significant.

**Table 4:** Association between attitude and knowledge.

Variables	Knowledge (Males)			Knowledge (Females)			Knowledge (Total)		
	n	Mean (±SD)	*p*-Value	n	Mean (±SD)	*p*-Value	n	Mean (±SD)	*p*-Value
A1. Do you agree that COVID-19 will finally be successfully controlled?									
Yes	134	9.49 (2.12)	NS	118	10.16 (1.80)	0.003	252	9.80 (2.00)	0.011
No/Unsure	37	9.16 (2.87)		31	8.87 (2.93)		68	9.03 (2.88)	
A2. Do you think that COVID-19 is a threat for your community?									
Yes	114	9.76 (1.88)	0.005	104	10.29 (1.52)	<0.001	218	10.01 (1.74)	<0.001
No/Unsure	57	8.72 (2.85)		45	8.98 (2.96)		102	8.83 (2.89)	
A3. I think that the lockdown would improve the overall wellbeing of the society in terms of controlling COVID-19 pandemic situation?									
Yes	148	9.72 (1.82)	<0.001	138	10.21 (1.52)	<0.001	286	9.96 (1.70)	<0.001
No/Unsure	23	7.43 (3.73)		11	5.91 (4.13)		34	6.94 (3.87)	

Note: independent samples *t*-test was used.

**Table 5:** Gender practices toward COVID-19 (n = 320).

Item	Male (%)	Female (%)	Total (%)	*p* Value *
P1. In recent days, have you gone to any crowded place?				0.04
No	79 (46.2)	86 (57.7)	165 (51.6)	
Yes/Unsure	92 (53.8)	63 (42.3)	155 (48.4)	
P2. In recent days, have you worn a mask when leaving home?				NS
Yes	141 (82.5)	131 (87.9)	272 (85.0)	
No/Unsure	30 (17.5)	18 (12.1)	48 (15.0)	
P3. Are you following the strategies recommended by authorities (e.g., Ministry of Health) to prevent the infection and spread of COVID-19?				0.01
Yes	136 (79.5)	134 (89.9)	270 (84.4)	
No/Unsure	35 (20.5)	15 (10.1)	50 (15.6)	

* chi-square test; NS—not significant.

**Table 6:** Association between practice and knowledge.

Variables	Knowledge (Males)			Knowledge (Females)			Knowledge (Total)		
	n	Mean (±SD)	*p*-Value	n	Mean (±SD)	*p*-Value	n	Mean (±SD)	*p*-Value
P1. In recent days, have you gone to any crowded place?									
No	79	9.80 (1.76)	0.04	86	10.00 (1.80)	NS	165	9.90 (1.83)	0.028
Yes/Unsure	92	9.09 (2.64)		63	9.75 (2.44)		155	9.35 (2.57)	
P2. In recent days, have you worn a mask when leaving home?									
Yes	141	9.76 (1.84)	<0.001	131	10.11 (1.61)	0.001	272	9.93 (1.74)	<0.001
No/Unsure	30	7.80 (3.36)		18	8.28 (4.11)		48	7.98 (3.62)	
P3. Are you following the strategies recommended by authorities (e.g., Ministry of Health) to prevent the infection and spread of COVID-19?									
Yes	136	9.71 (1.89)	0.001	134	10.05 (1.82)	0.006	270	9.88 (1.86)	<0.001
No/Unsure	35	8.29 (3.26)		15	8.47 (3.80)		50	8.34 (3.39)	

Note: independent samples *t*-test was used.

## Data Availability

The datasets used for this study were available from the corresponding author on reasonable request.

## References

[1] World Health Organization Novel Coronavirus (2019-nCoV) Situational Report-11. https://www.who.int/docs/default-source/coronaviruse/situation-reports/20200121-sitrep-1-2019-ncov.pdf.

[2] Zhou P., Yang X.L., Wang X.G., Hu B., Zhang L., Zhang W., Si H.R., Zhu Y., Li B., Huang C.L. (2020). A pneumonia outbreak associated with a new coronavirus of probable bat origin. Nature.

[3] Wu F., Zhao S., Yu B., Chen Y.M., Wang W., Song Z.G., Hu Y., Tao Z.W., Tian J.H., Pei Y.Y. (2020). A new coronavirus associated with human respiratory disease in China. Nature.

[4] World Health Organization Novel Coronavirus (2019-nCoV) Situational Report-11. https://apps.who.int/iris/bitstream/handle/10665/330776/nCoVsitrep31Jan2020-eng.pdf?sequence=1&isAllowed=y.

[5] World Health Organization Coronavirus Disease (COVID-19) Pandemic. https://www.euro.who.int/en/health-topics/health-emergencies/coronavirus-covid-19/novel-coronavirus-2019-ncov.

[6] Ali M.Y., Gatiti P. (2020). The COVID-19 (Coronavirus) pandemic: Reflections on the roles of librarians and information professionals. Health Inf. Libr. J..

[7] Riou J., Althaus C.L. (2020). Pattern of early human-to-human transmission of Wuhan 2019 novel coronavirus (2019-nCoV), December 2019 to January 2020. Eurosurveillance.

[8] Chan J.F., Yuan S., Kok K.H., To K.K., Chu H., Yang J., Xing F., Liu J., Yip C.C., Poon R.W. (2020). A familial cluster of pneumonia associated with the 2019 novel coronavirus indicating person-to-person transmission: A study of a family cluster. Lancet.

[9] Chen N., Zhou M., Dong X., Qu J., Gong F., Han Y., Qiu Y., Wang J., Liu Y., Wei Y. (2020). Epidemiological and clinical characteris-tics of 99 cases of 2019 novel coronavirus pneumonia in Wuhan, China: A descriptive study. Lancet.

[10] Ministry of Health (MOH) MOH Reports First Case of Coronavirus Infection. https://www.moh.gov.sa/en/Ministry/MediaCenter/News/Pages/News-2020-03-02-002.aspx.

[11] Alrasheedy A.A., Abdulsalim S., Farooqui M., Alsahali S., Godman B. (2021). Knowledge, attitude and practice about coronavirus dis ease (COVID-19) pandemic and its psychological impact on students and their studies: A cross-sectional study among pharmacy students in Saudi Arabia. Risk Manag. Healthc. Policy.

[12] Turoń K., Kubik A. (2021). Business innovations in the new mobility market during the COVID-19 with the possibility of open business model innovation. J. Open Innov. Technol. Mark. Complex..

[13] Chirwa G.C. (2020). “Who knows more, and why?” Explaining socioeconomic related inequality in knowledge about HIV in Malawi. Sci. Afr..

[14] Chirwa G.C. (2019). Socio-economic Inequality in Comprehensive Knowledge about HIV in Malawi. Malawi Med. J..

[15] Podder D., Paul B., Dasgupta A., Bandyopadhyay L., Pal A., Roy S. (2019). Community perception and risk reduction practices toward malaria and dengue: A mixedmethod study in slums of Chetla, Kolkata. Ind. J. Public Health.

[16] Amarie A., Udijono A., Kusariana N., Saraswati L.D. (2020). Description of Knowledge, Attitude, and Practice of Coronavirus Disease-19 Prevention Based on Gender and Age in Java Island Community. J. Public Health Trop. Coast. Region..

[17] Alhajjaj A.H., Aldarweesh H.H., Alghawi Z.A. (2020). Knowledge, Attitude, and Awareness Related to COVID-19 Pandemic among the Public, Saudi Arabia: A Cross-Sectional Descriptive Study. Eur. J. Med. Educ. Technol..

[18] Almoayad F.A., Bin-Amer L.A., Mahboub S., Alrabiah A.M., Alhashem A.M. (2021). Preventive practices against COVID-19 among residents of Riyadh, Saudi Arabia. J. Infect. Dev. Ctries..

[19] Al-Hanawi M.K., Angawi K., Alshareef N., Qattan A., Helmy H.Z., Abudawood Y., Alqurashi M., Kattan W.M., Kadasah N.A., Chirwa G.C. (2020). Knowledge, attitude and practice toward COVID-19 among the public in the Kingdom of Saudi Arabia: A cross-sectional study. Front. Public Health.

[20] Raosoft Sample Size Calculator 2020. http://www.raosoft.com/samplesize.html.

[21] Al-Wutayd O., Mansour A.E., Aldosary A.H., Hamdan H.Z., Al-Batanony M.A. (2021). Handwashing knowledge, attitudes, and prac-tices during the COVID-19 pandemic in Saudi Arabia: A non-representative cross-sectional study. Sci. Rep..

[22] STATS General Authority for Statistics: The Kingdom of Saudi Arabia 2020. https://www.stats.gov.sa/en/node.

[23] Bland J.M., Altman D.G. (1997). Statistics notes: Cronbach’s alpha. BMJ.

[24] Hezima A., Aljafari A., Aljafari A., Mohammad A., Adel I. (2020). Knowledge, attitudes, and practices of Sudanese residents towards COVID-19. East Mediterr. Health J..

[25] Mousa K.N., Saad M.M., Abdelghafor M.T. (2020). Knowledge, attitudes, and practices surrounding COVID-19 among Sudan citizens during the pandemic: An online cross-sectional study. Sudan J. Med. Sci..

[26] Tawalbeh L.I., Al-Smadi A.M., Ashour A., Alshajrawi A., Gammoh O., Abu-AL-Rous N. (2021). Public knowledge, attitudes and prac-tice about COVID-19 pandemic. J. Public Health Afr..

[27] Azlan A.A., Hamzah M.R., Sern T.J., Ayub S.H., Mohamad E. (2020). Public knowledge, attitudes and practices towards COVID-19: A cross-sectional study in Malaysia. PLoS ONE.

[28] Shukla S., Deotale P. (2020). Knowledge, attitude and practices towards COVID-19 pandemic in the community: A cross-sectional web-based survey in India. Int. J. Res. Med. Sci..

[29] Kasemy Z.A., Bahbah W.A., Zewain S.K., Haggag M.G., Alkalash S.H., Zahran E., Desouky D.E. (2020). Knowledge, Attitude and Practice toward COVID-19 among Egyptians. J. Epidemiol. Glob. Health.

[30] Rahman S.M., Akter A., Mostari K.F., Ferdousi S., Ummon I.J., Naafi S.M., Rahman M.M., Uddin M.G., Tasmin S., Uddin M.A. (2020). Assessment of knowledge, attitudes and practices towards prevention of coronavirus disease (COVID-19) among Bang-ladeshi population. Bangladesh Med. Res. Counc. Bull..

[31] Kundu S., Al Banna M., Sayeed A., Begum M.R., Brazendale K., Hasan M.T., Habiba S.J., Abid M.T., Khan M., Chowdhury S. (2021). Knowledge, attitudes, and preventive practices toward the COVID-19 pandemic: An online survey among Bangladeshi residents. J. Public Health.

[32] Habib M.A., Dayyab F.M., Iliyasu G., Habib A.G. (2021). Knowledge, attitude and practice survey of COVID-19 pandemic in Nortern Nigeria. PLoS ONE.

[33] Wilson J.B., Deckert A., Shah R., Kyei N., Dahn L.C., Doe-Rogers R., Hinneh A.B., Johnson L.W., Natt G.D., Verdier J.A. (2021). COVID-19-related knowledge, attitudes and practices: A mixed-mode cross-sectional survey in Liberia. BMJ Open.

[34] Ngwewondo A., Nkengazong L., Ambe L.A., Ebogo J.T., Mba F.M., Goni H.O., Nyunaï N., Ngonde M.C., Oyono J.E. (2020). Knowledge, attitudes, practices of/towards COVID 19 preventive measures and symptoms: A cross-sectional study during the exponen-tial rise of the outbreak in Cameroon. PLoS Negl. Trop. Dis..

[35] Alnasser A.H., Al-Tawfiq J.A., Al-Kalif M.S., Shahadah R.F., Almuqati K.S., Al-Sulaiman B.S., Alharbi K.K., Alabbad F.Y., Alabbad J.Y., Alquwaiz I.A. (2021). Public knowledge, attitudes, and practice towards COVID-19 pandemic in Saudi Arabia: A web-based cross-sectional survey. Med. Sci..

[36] Gao H., Hu R., Yin L., Yuan X., Tang H., Luo L., Chen M., Huang D., Wang Y., Yu A. (2020). Knowledge, attitudes and practices of the Chinese public with respect to coronavirus disease (COVID-19): An online cross-sectional survey. BMC Public Health..

[37] Okello G., Izudi J., Teguzirigwa S., Kakinda A., Van Hal G. (2020). Findings of a cross-sectional survey on knowledge, attitudes, and practices about COVID-19 in Uganda: Implications for public health prevention and control measures. BioMed Res. Int..

[38] Rabbani M.G., Akter O., Hasan M.Z., Samad N., Mahmood S.S., Joarder T. (2020). Knowledge, Attitude and Practice towards COVID-19 among people in Bangladesh during the pandemic: A cross-sectional study. medRxiv.

[39] Feleke B.T., Wale M.Z., Yirsaw M.T. (2021). Knowledge, attitude and preventive practice towards COVID-19 and associated factors among outpatient service visitors at Debre Markos compressive specialized hospital, north-west Ethiopia, 2020. PLoS ONE.

[40] Begum F. (2020). Knowledge, attitudes, and practices towards covid-19 among B. Sc. Nursing students in selected nursing institution in Saudi Arabia during COVID-19 outbreak: An online survey. Saudi J. Nurs. Health Care..

[41] Rugarabamu S., Ibrahim M., Byanaku A. (2020). Knowledge, attitudes, and practices (KAP) towards COVID-19: A quick online cross-sectional survey among Tanzanian residents. BMJ.

[42] Haque T., Hossain K.M., Bhuiyan M.M., Ananna S.A., Chowdhury S.H., Islam M.R., Ahmed A., Rahman M.M. (2020). Knowledge, Attitude and Practices (KAP) towards COVID-19 and Assessment of Risks of Infection by SARS-CoV-2 among the Bangladeshi Population: An Online cross Sectional Survey. Res. Sq..

[43] Sultana M.S., Khan A.H., Islam M.R., Hossain S., Hasan M.T., Kurasaki M., Sikder M.T. (2020). Gender differences in Knowledge, Attitude and Preparedness to Respond to COVID-19 among adult population in Bangladesh: A Cross-sectional Study. Preprints.

[44] Al-Atiyyat N., Al-Rawashdeh S., Mrayyan M.T. (2021). A Web-Based Study of Differences in Jordanian People’s Knowledge and Attitudes toward COVID-19. Electr. J. Gen. Med..

[45] Yang K., Liu H., Ma L., Wang S., Tian Y., Zhang F., Li Z., Song Y., Jiang X. (2021). Knowledge, attitude and practice of residents in the prevention and control of COVID-19: An online questionnaire survey. J. Adv. Nurs..

[46] Tawiah P.A., Arhin-Wiredu K., Oppong K., Torgbor B.N., Konadu P.E., Abaka-Yawson A. (2021). Socio-demographic characteristics influencing knowledge, attitude and preventive practices of COVID-19 among Ghanaians: A cross-sectional study. Ann. Antivir. Antiretrovir..

[47] Khan Z.H., Manzoor I., Qureshi I. (2020). Gender Differences In Knowledge, Attitude And Perceptions Re-garding Covid-19 among General Population Of Punjab, Pakistan. Br. J. Pharm. Med..

[48] Zhong B.L., Luo W., Li H.M., Zhang Q.Q., Liu X.G., Li W.T., Li Y. (2020). Knowledge, attitudes, and practices towards COVID-19 among Chinese residents during the rapid rise period of the COVID-19 outbreak: A quick online cross-sectional survey. Int. J. Biol. Sci..

[49] Reuben R.C., Danladi M.M., Saleh D.A., Ejembi P.E. (2021). Knowledge, attitudes and practices towards COVID-19: An epidemiological survey in North-Central Nigeria. J. Community Health.

[50] Yue S., Zhang J., Cao M., Chen B. (2020). Knowledge, attitudes and practices of COVID-19 among urban and rural residents in china: A cross-sectional study. J. Community Health.

[51] Abbasi-Kangevari M., Kolahi A.A., Ghamari S.H., Hassanian-Moghaddam H. (2021). Public Knowledge, Attitudes, and Practices Re-lated to COVID-19 in Iran: Questionnaire Study. JMIR Public Health Surveill..

[52] Almutairi A.F., BaniMustafa A.A., Alessa Y.M., Almutairi S.B., Almaleh Y. (2020). Public trust and compliance with the precautionary measures against COVID-19 employed by authorities in Saudi Arabia. Risk Manag. Health Policy.

[53] Alahdal H., Basingab F., Alotaibi R. (2020). An analytical study on the awareness, attitude and practice during the COVID-19 pandemic in Riyadh, Saudi Arabia. J. Infect. Public Health.

[54] Pal R., Yadav U., Grover S., Saboo B., Verma A., Bhadada S.K. (2020). Knowledge, attitudes and practices towards COVID-19 among young adults with Type 1 diabetes mellitus amid the nationwide lockdown in India: A cross-sectional survey. Diabetes Res. Clin. Pract..

[55] Kartheek A., Gara K., Vanamali D. (2020). Knowledge, attitude and practices towards COVID-19 among Indian residents during the pandemic: A cross-sectional online survey. J. NTR Univ. Health Sci..

[56] Ali A., Farooq S., Khalid N., Ahmed F. (2020). Awareness, attitude and practices related to COVID-19 pandemic in General Public of Province Sindh, Pakistan. Pak. J. Med. Dent..

[57] Hussain A., Garima T., Singh B., Ram R., Tripti R. (2020). Knowledge, attitudes, and practices towards COVID-19 among Nepalese Residents: A quick online cross-sectional survey. AJMS.

[58] Galasso V., Pons V., Profeta P., Becher M., Brouard S., Foucault M. (2020). Gender differences in COVID-19 attitudes and behavior: Panel evidence from eight countries. Proc. Natl. Acad. Sci. USA.

[59] Pawlowski B., Atwal R., Dunbar R. (2008). Sex Differences in Everyday Risk-Taking Behavior in Humans. Evol. Psychol..

[60] Cobey K.D., Stulp G., Laan F., Buunk A.P., Pollet T.V. (2013). Sex differences in risk taking behavior among Dutch cyclists. Evol. Psychol..

[61] Duell N., Steinberg L., Icenogle G., Chein J., Chaudhary N., Di Giunta L., Dodge K.A., Fanti K.A., Lansford J.E., Oburu P. (2018). Age patterns in risk taking across the world. J. Youth Adolesc..

[62] Jadoo S.A., Danfour O.M., Zerzah M., Abujazia M.A., Torun P., Al-Samarrai M.A., Yaseen S.M. (2021). Knowledge, attitude, and practice towards COVID-19 among Libyan people-a web-based cross-sectional study. J. Ideas Health.

[63] AlRasheed M.M., AlShahrani A.H., AlMuhaini S.A., AlKofide H.A., Alhawassi T.M., Aldemerdash A., Alhaj O.A., Bragazzi N.L., Jahrami H.A. (2021). Knowledge, attitude, and practice towards COVID-19 among pharmacists: A cross-sectional study. Risk Manag. Healthc. Policy.

[64] Nyeko R., Amanya S.B., Aleni M., Akello F. (2021). Unsatisfactory COVID-19-related knowledge, attitudes and practices among undergraduate university students in Uganda: An online cross-sectional survey. Open J. Prev. Med..

